# The Green Lean Amine Machine: Harvesting Electric Power While Capturing Carbon Dioxide from Breath

**DOI:** 10.1002/advs.202100995

**Published:** 2021-05-28

**Authors:** Trevor J. Kalkus, Anirvan Guha, Philip B.V. Scholten, Dmitrii Nagornii, Ali Coskun, Alessandro Ianiro, Michael Mayer

**Affiliations:** ^1^ Adolphe Merkle Institute University of Fribourg Chemin des Verdiers 4 Fribourg 1700 Switzerland; ^2^ Radboud University Houtlaan 4 Nijmegen 6525 XZ Netherlands; ^3^ Department of Chemistry University of Fribourg Chemin du Musee 9 Fribourg 1700 Switzerland

**Keywords:** breath, carbon capture, monoethanolamine, reverse electrodialysis

## Abstract

As wearable technologies redefine the way people exchange information, receive entertainment, and monitor health, the development of sustainable power sources that capture energy from the user's everyday activities garners increasing interest. Electric fishes, such as the electric eel and the torpedo ray, provide inspiration for such a power source with their ability to generate massive discharges of electricity solely from the metabolic processes within their bodies. Inspired by their example, the device presented in this work harnesses electric power from ion gradients established by capturing the carbon dioxide (CO_2_) from human breath. Upon localized exposure to CO_2_, this novel adaptation of reverse electrodialysis chemically generates ion gradients from a single initial solution uniformly distributed throughout the device instead of requiring the active circulation of two different external solutions. A thorough analysis of the relationship between electrical output and the concentration of carbon capture agent (monoethanolamine, MEA), the amount of CO_2_ captured, and the device geometry informs device design. The prototype device presented here harvests enough energy from a breath‐generated ion gradient to power small electronic devices, such as a light‐emitting diode (LED).

## Graphical abstract

Carbon capture technology is combined with reverse electrodialysis to harvest energy from an ion gradient generated by the reaction of monoethanolamine with carbon dioxide in human breath. Several factors are characterized that affect power output, and ultimately this work demonstrates the ability to power an light‐emitting diode with the ion gradient generated by breath.

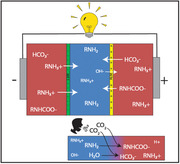

## 1 Introduction

Wearable technologies are redefining the ways by which humans exchange information, receive entertainment, and monitor health and fitness.^[^ ^1^, ^2^ ^]^ Although traditional electrochemical batteries are available to power these electronics, their limited lifetime and incompatibility with biology are not ideal for powering devices that interface with the human body. It is therefore compelling to explore novel power sources that can be fueled by the activity of the wearer. Without needing to be “plugged in”, these power sources could integrate seamlessly into daily life. The knifefish of the genus *Electrophorus*, commonly known as electric eels, provide a potent example of the ability to generate electricity from metabolic energy. Electric eels use the flux of ions across the membranes of their electrically‐active cells (known as “electrocytes”) to produce external electrical discharges of over 800 V and 1 A.^[^ ^3^, ^4^ ^]^ Within the electric organs of electric eels, arrays of thousands of electrocytes fire simultaneously to generate each electric discharge.

Previously, inspired by electric eels, we developed a soft, potentially biocompatible power source that represented the first step toward a novel approach for powering implantable electronics.^[^ ^5^ ^]^ This “artificial electric organ” stacked a repeat sequence of high‐salinity, cation‐selective, low‐salinity, and anion‐selective hydrogels in series to produce over 100 V from ion gradients. Unlike the electric eel, however, this system was unable to re‐establish its ion gradients without an external power source. A similar bioinspired device that can restore and maintain its ion gradients by utilizing natural metabolic processes would represent another step toward fully realizing the electric eels’ power generation capabilities. Portable, wearable, and possibly even implantable electrical power sources charged by natural metabolic processes could lead to a paradigm shift for powering electronics.

Existing power sources capable of harvesting energy from human activity and metabolism are often too intrusive to be practical for general purposes. For example, some devices oxidize glucose, an important chemical source of energy found in the bloodstream, to generate electrical power. Impressively, this reaction can drive self‐powered glucose monitors.^[^ ^6^, ^7^, ^8^ ^]^ Another power source demonstrated the ability to use ion concentration gradients maintained by the inner ear to power a radio signal.^[^ ^9^ ^]^ These examples are best suited for addressing the low‐power needs of inherently invasive devices (e.g., diagnostic devices that necessarily require access to blood or other internal body fluids) rather than general applications. Alternatively, piezoelectric power sources that generate energy from the user's movement provide an example of a noninvasive wearable power source.^[^ ^10^ ^]^ Additionally, Jia et al. used the oxidation of lactate in sweat, which can be accessed noninvasively, to fuel a wearable power source that illuminated a light‐emitting diode (LED) and a digital watch.^[^ ^11^ ^]^ Although these power sources are noninvasive, they both require a certain level of physical activity that the user cannot maintain at all times.

Exhaled breath and its carbon dioxide (CO_2_) content provide a continuous and noninvasively accessible waste product of human metabolism, presenting a compelling candidate as a fuel for wearable power sources. Xue et al. developed a noninvasive wearable pyroelectric nanogenerator built into a surgical face mask that scavenged energy from the temperature fluctuation of human breath.^[^ ^12^ ^]^ No power source, however, has used the continuous availability of CO_2_ supplied by human breath as its fuel.

The development of power sources that use CO_2_ as “fuel” has garnered interest among the scientific community aiming to increase sustainability and reduce the environmental footprint of industrial processes.^[^ ^13^, ^14^, ^15^, ^16^, ^17^, ^18^, ^19^ ^]^ Kim et al. recently applied reverse electrodialysis (RED) to convert electric power from the difference in ion concentration between deionized water and an amine solution used to capture CO_2_, which they referred to as carbon capture RED (CCRED).^[^ ^19^ ^]^ Similar to the eel‐inspired power source,^[^ ^5^ ^]^ RED harvests energy from ion concentration gradients to generate electricity. Along with pressure retarded osmosis, RED is one of the two primary methods for harvesting salinity‐gradient power.^[^ ^20^, ^21^, ^22^, ^23^ ^]^ A typical RED device consists of alternating cation‐ and anion‐selective membranes that separate alternating compartments of high and low ionic strength. Cations diffuse from the high ionic strength solution through the cation exchange membrane (CEM) in one direction, while anions diffuse from the high ionic strength solution through the anion exchange membrane (AEM) in the opposite direction. This directed flux of ions creates an additive electromotive force across each selective membrane, which is converted into electrical power by reduction‐oxidization reactions at electrodes on both ends of the RED apparatus.^[^ ^24^, ^25^, ^26^, ^27^ ^]^ Extensive experimental and theoretical work provides the background for the optimization of RED technology to promote access to the vast amount of energy available from the mixing of freshwater with seawater that occurs constantly.^[^ ^26^, ^27^, ^28^, ^29^ ^]^ Using this foundation, the internal resistance of RED power sources have been reduced by minimizing the intermembrane distance and by using specialized ion exchange membranes to reach power densities up to 2.9 W m^−2^.^[^ ^30^, ^31^, ^32^ ^]^ Using brines or wastewater as the high ionic strength solution instead of seawater allows access to even higher power densities.^[^ ^33^ ^]^ Upscaling RED technology presents challenges that include decreased power density due to the increased residence time of the solutions in large compartments and the fouling of ion exchange membranes over time when using natural solutions.^[^ ^34^, ^35^, ^36^, ^37^ ^]^ Nonetheless, researchers have developed pilot RED power plants designed for freshwater and seawater/brines/wastewater, with Tedesco et al. aiming to reach and maintain a power output of 1 kW.^[^ ^36^, ^37^, ^38^ ^]^ In nearly all implementations of RED, the constant flow of the two solutions maintains the difference in ionic strength between adjacent compartments and thus allows these devices to provide electrical power continuously. One creative and inspiring RED design, dubbed “precipitation‐assisted solid salt RED,” used salts that dissolve into the high ionic strength solution and precipitate out of the low ionic strength solution.^[^ ^39^ ^]^ This system generated an ion gradient without the requirement of pumps, an elegant strategy that inspired the design presented in this work.

The carbon capture RED (CCRED) work introduced by Kim et al. demonstrated that a CO_2_‐rich solution can be used as the high ionic strength solution instead of seawater due to the generation of bicarbonate and protons, two oppositely‐charged species, during the carbon capture process.^[^ ^19^ ^]^ Kim et al. chose *N*‐methyldiethanolamine (MDEA) as the carbon capture agent in their CCRED device partly because of the low temperature required to release the captured CO_2_ and regenerate the original amine solution for repeated use.^[^ ^19^ ^]^ To help limit large‐scale CO_2_ emissions that contribute to climate change,^[^ ^40^ ^]^ carbon capture research primarily uses chemical agents, often amines, that react readily with CO_2_.^[^ ^41^, ^42^, ^43^, ^44^ ^]^ The energy required to regenerate the aqueous amine solution following CO_2_ capture is an important consideration for industrial‐scale applications where the same solution is recycled and reused.^[^ ^45^, ^46^ ^]^ Conversely, for the purpose of capturing CO_2_ from breath that has a low carbon content compared to exhaust from industrial processes (≈3.2–6.5 mol% CO_2_ in exhaled breath^[^ ^47^ ^]^ compared to 12–15 mol% in coal‐based power plants and 20–44 mol% in iron production^[^ ^48^ ^]^), the use of a fast reacting capture agent is advantageous. Monoethanolamine (MEA) is considered the benchmark for solvent‐based carbon capture because of its rapid reaction kinetics with CO_2_.^[^ ^41^, ^42^, ^49^ ^]^ In work related to CCRED, Hamelers et al. used carbon capture with a 1.5 wt% MEA solution to generate a power density of 4.5 mW m^−2^ with capacitive electrodes, demonstrating the compatibility of this carbon capture solution with electricity‐generating techniques that employ charge‐selective membranes.^[^ ^17^, ^50^, ^51^ ^]^

**Figure 1:**
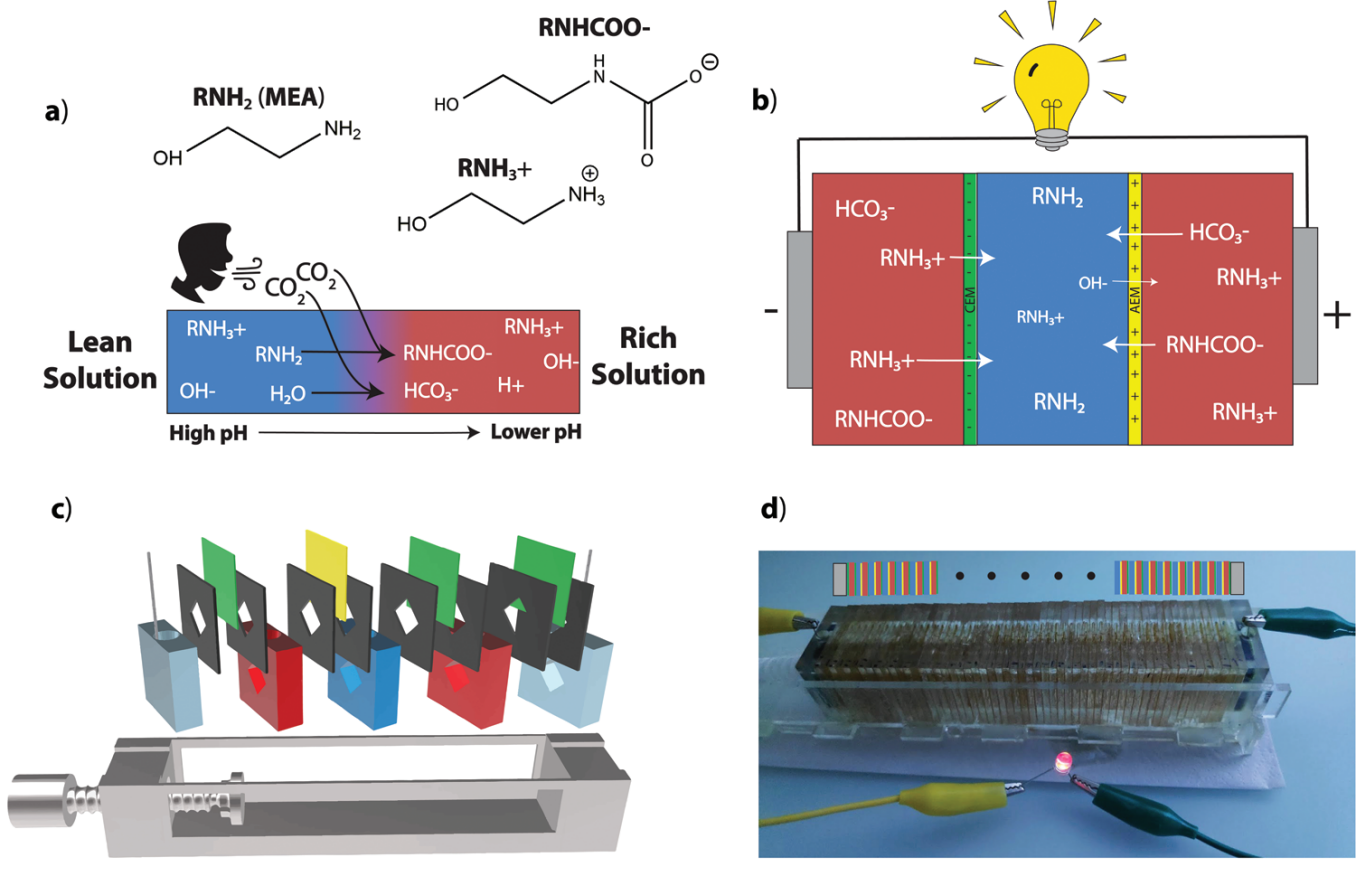
Principle of using breath‐generated ion gradients to harness electrical power. a) The addition of CO₂ from breath to a lean MEA solution results in a reaction that lowers the pH and generates charged species. The chemical structure of MEA and the ions that result from the reaction of CO₂ with MEA are drawn. b) Diagram of a single electricity‐generating cell. A cation exchange membrane (CEM) and an anion exchange membrane (AEM) separate the lean solution compartment from the rich solution compartments. Cations (RNH₃+) diffuse down their ion gradient from rich to lean across the CEM. Anions (RNHCOO—, HCO₃—) diffuse from rich to lean across the AEM. Hydroxide diffuses from the lean to rich across the AEM. Electrodes at either end convert ionic current into electric current. c) Cartoon of prototype device, color‐coded to match the diagram in panel b). The additional CEMs on either end contain the anionic hexacyanoferrate (HCF) solution within the electrode compartments. Openings at the top of the electrode compartments allow access for the platinum wires and for refreshing the HCF solution. Similarly, each other compartment also has an opening at the top for exchanging solutions. The black components represent the neoprene gaskets used to create a seal around the membranes. The diamond openings (1 cm^2^) in the compartments and gaskets hold the lean and rich solutions, which are contained by the membranes. We used a diamond orientation instead of a square to help facilitate the escape of gas bubbles out of the top of each compartment. In some cases, including the demonstration in panel d, we used vacuum grease in the place of neoprene gaskets. d) Picture of a LED powered by ionic gradients established using breath supplied to a stack of 27 RED cells in series. The pattern above the device indicates the components of the repeating cells using the same color‐coding from panels b) and c) (electrode compartments: gray, rich compartments: red, lean compartments: blue, CEM: green, AEM: yellow). The black dots indicate that the pattern continues to repeat.

Here we present a CCRED prototype that harvests electrical power from the ion gradient generated by capturing CO_2_ in human breath. Lean solution (MEA solution before the addition of CO_2_) serves as the low ionic strength solution, and the addition of CO_2_ results in the formation of carbamate, bicarbonate, and protonated MEA ions, generating the “rich” solution that served as the high ionic strength solution (**Figure** **1**a,b). The reaction causes the pH of the MEA solution to decrease, allowing carbon loading (the amount of CO_2_ captured by the MEA solution) to be monitored by measuring pH. Figure 1b illustrates the movement of ions across the selective membranes for a device with one RED unit cell. Each of these unit cells, consisting of a rich compartment, a CEM, a lean compartment, and an AEM, increases the potential of the device linearly when added in series (Figure S1, Supporting Information). We used 3D printing to rapidly create the prototype design (Figure 1c) that allowed for the straightforward exchange of solutions and the ability to adjust the number of cells in series. Using this design, we evaluated the relationship between power output and several device characteristics: MEA concentration, carbon loading, and device geometry. Ultimately, we demonstrated that the electrical power harvested from ion gradients generated by capturing CO_2_, both from pure CO_2_ gas as well as breath, can power a light‐emitting diode (LED) (Figure 1d). Unlike most previous RED devices that require the exchange of external solutions to establish an ion gradient, we demonstrated the ability to generate an ion gradient in situ via the addition of CO_2_ to alternating compartments. In this case, prior to the addition of CO_2_, the same lean MEA solution filled all the compartments and the system remained in thermodynamic equilibrium. This design requires only one uniform initial solution and eliminates the need for fluidic pumps, representing novel advantages over most other RED designs.

## 2 Optimization of the MEA Concentration

**Figure 2:**
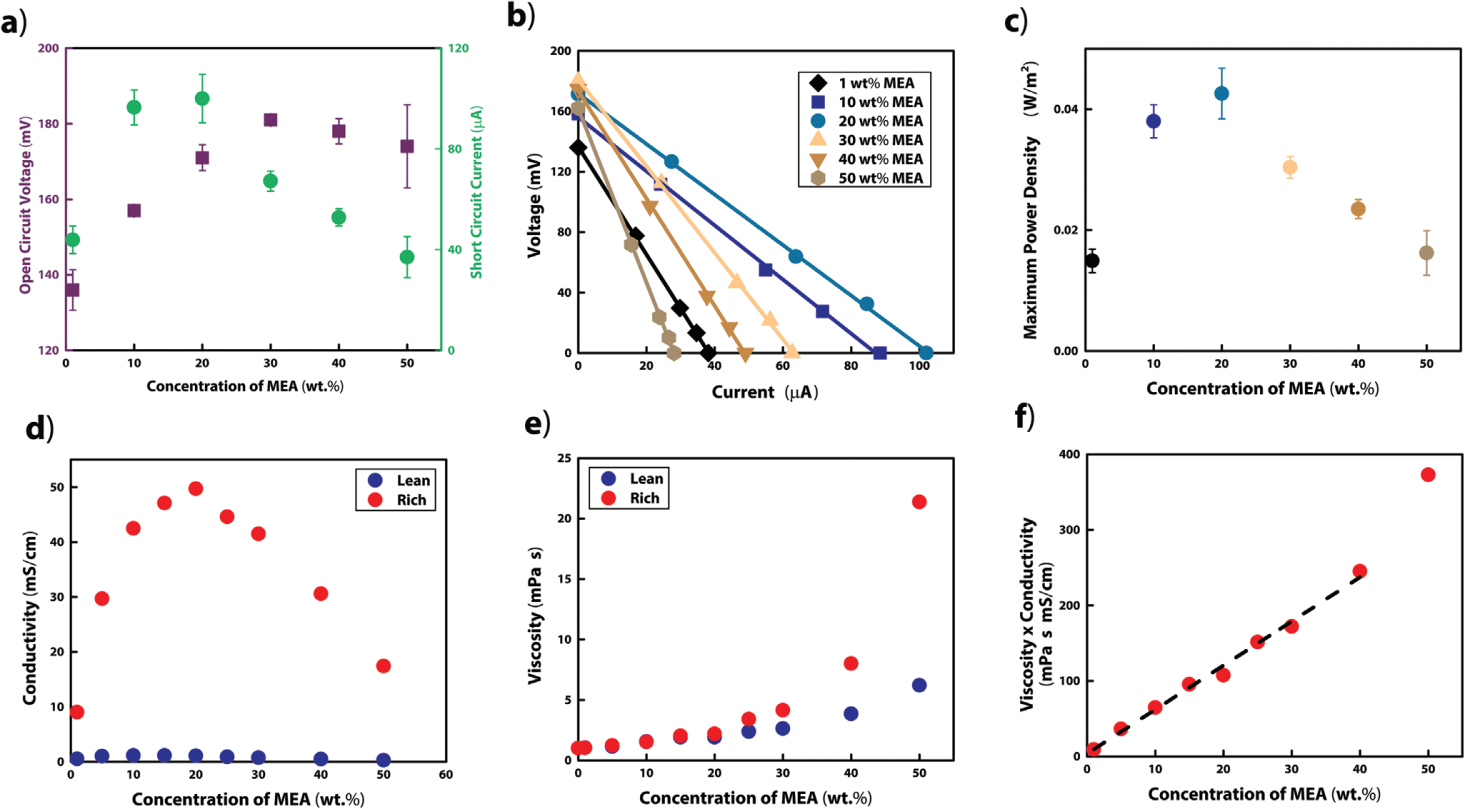
Optimization of MEA concentration for power output. a) Measured *V*_oc_ and *I*_sc_ at varied MEA concentrations using rich solutions at maximum carbon loading (mean ± SD, *n* = 5 except at 20 wt% where *n* = 6). b) Representative *I*–*V* curves of the device when using different MEA concentrations. All lines are linear fits. c) Maximum power densities at varied concentrations of MEA, calculated using *V*_oc_ and *I*_sc_ measurements in panel a). d) Conductivity of lean and rich solutions as a function of MEA concentration. e) Viscosity of lean and rich solutions as a function of MEA concentration. f) Plotting the product of viscosity and conductivity of the rich solution as a function of concentration confirmed Equation (1) and supported the hypothesis that increased viscosity resulted in decreased power density at high MEA concentrations.

To optimize the power output of the CCRED device presented here, we evaluated its electrical characteristics across a range of MEA concentrations. For each MEA concentration, we added CO_2_ to the rich solution until the pH stabilized, indicating that the solution reached maximum carbon loading. To analyze power, we measured the open circuit voltage (*V*_oc_), the potential when current approaches zero, and the short circuit current (*I*_sc_), the current when voltage approaches zero (**Figure** **2**a). We used these measurements to construct the linear current–voltage curves (*I*–*V* curves) that are characteristic of RED devices and the electrophysiology of the electric eel (Figure 2b)^[^ ^5^, ^52^ ^]^ and to calculate the maximum power density (Figure 2c; and Equation S7, Supporting Information). We found that 20 wt% MEA demonstrated the peak maximum power density (43 ± 4 mW m^−2^) largely due the relatively high current output of RED cells filled with this solution. To understand this finding, we measured the conductivity (*κ*) of solutions with different MEA concentrations and found that the peak *κ* occurred at approximately 20 wt% MEA for the rich solution and 15 wt% MEA for the lean solution (Figure 2d). The conductivity of a solution (*κ*) is proportional to the ratio between the concentration of free charge carriers within a solution ([*C*]) and that solution's viscosity (*η*)^[^ ^53^ ^]^

(1) $$\kappa \propto \frac{\left[C\right]}{\eta }$$

Although we expected [*C*] to increase proportionally with MEA concentration, we found that *η* increases nonlinearly with increasing MEA concentration (Figure 2e). In Figure 2f, we correlated *η, κ*, and MEA concentration to confirm this relationship and support the hypothesis that the observed decrease in conductivity at high MEA concentrations resulted from increased viscosity. The measurements taken with 50 wt% MEA solution did not follow this linear trend, likely due to nonrandom mixing of the ionic species at high ionic concentrations.^[^ ^54^ ^]^ The high conductivity of the 20 wt% MEA solution resulted in a lower internal resistance and a higher current output compared to using solutions with lower or higher MEA concentrations.

Research in the field of RED often reports that the low ionic‐strength compartment provides the highest contribution to the internal resistance of an RED device.^[^ ^55^ ^]^ By using a lean solution (at 20 wt% MEA, *κ* = 1.1 mS cm^−1^) instead of deionized water^[^ ^19^ ^]^ (*κ* = 5.5 × 10^−5^ mS cm^−1^), we greatly reduced the internal resistance of the low ionic‐strength compartments (Figure S2, Supporting Information). We proceeded to use a 20 wt% MEA solution for all other experiments.

## 3 Relationship between Carbon Loading and Electric Potential

**Figure 3:**
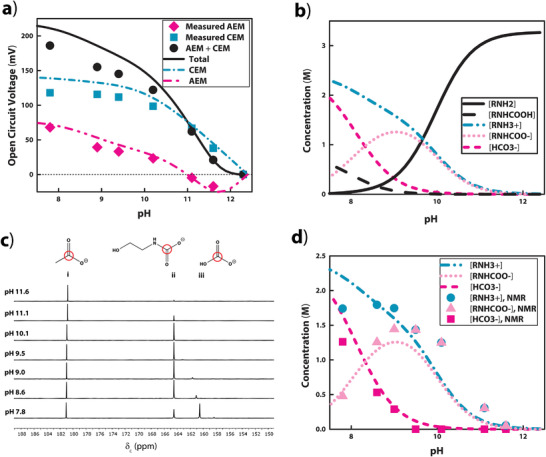
Carbon loading, as indicated by pH, in relation to the *V*_oc_ of the device and the concentration of ionic species. a) The data points represent the contribution of each membrane, CEM and AEM, and the total potential of a single RED unit as a function of the pH of the rich solution. The lean solution always remains at pH 12.2. The curves represent the predicted potential of each membrane and total potential using the theoretical model (Section S4 and Script S1, Supporting Information). b) Theoretically predicted concentrations of chemical species as a function of pH. The concentrations of CO_3_^2−^ and H_2_CO_3_ were negligible. c) The segment of data from ^13^C nuclear magnetic resonance spectroscopy measurements indicating i) acetate (standard), ii) carbamates, and iii) carbonates within MEA solutions across a range of pH values. We estimated carbamate and carbonate concentration using these data. The peaks correspond to the carbons circled in red on the drawn structures. d) Comparison between the concentrations of relevant charged chemical species calculated from the NMR spectra with those predicted by the mathematical model.

To examine the relationship between carbon loading and the electric potential of the device, we measured the *V*_oc_ across both the AEM and the CEM when the rich solution was loaded with varying amounts of CO_2_, as indicated by pH (**Figure** **3**a). The potential across each membrane (*V*_CEM_, *V*_AEM_) depends on the concentration of ionic species in the lean (*l*) and rich (*r*) compartments and the ability of each ionic species to cross the membrane, which is reflected by that membrane's relative permselectivity to that species ($P_{i}^{\mathrm{CEM}}\;\mathrm{and}\;P_{i}^{\mathrm{AEM}}$ for the ionic species *i* across the CEM and the AEM, respectively). When only monovalent ions are present, the Goldman–Hodgkin–Katz equation estimates the values of *V*_CEM_ and *V*_AEM_^[^ ^56^ ^]^

(2) $$\begin{array}{c} V_{\mathrm{CEM}}=\;\frac{RT}{F}\ln\left(\frac{\sum_{i-}P_{i-}^{\mathrm{CEM}}{\left[i-\right]}_{l}+\sum_{i+}P_{i+}^{\mathrm{CEM}}{\left[i+\right]}_{r}}{\sum_{i-}P_{i-}^{\mathrm{CEM}}{\left[i-\right]}_{r}+\sum_{i+}P_{i+}^{\mathrm{CEM}}{\left[i+\right]}_{l}\;}\right)\\ V_{\mathrm{AEM}}=\;\frac{RT}{F}\ln\left(\frac{\sum_{i-}P_{i-}^{\mathrm{AEM}}{\left[i-\right]}_{r}+\sum_{i+}P_{i+}^{\mathrm{AEM}}{\left[i+\right]}_{l}}{\sum_{i-}P_{i-}^{\mathrm{AEM}}{\left[i-\right]}_{l}+\sum_{i+}P_{i+}^{\mathrm{AEM}}{\left[i+\right]}_{r}\;}\right) \end{array}$$

where *R* is the universal gas constant (8.314 J mol^−1^ K^−1^), *T* is the absolute temperature (K), *F* is the Faraday constant (96 485 C mol^−1^), and *i* − and *i* + indicate anionic and cationic species, respectively. To apply Equation (2), we needed to estimate the concentration of ionic species as CO_2_ was added to the solution. We first modified a theoretical model developed by McCann et al.^[^ ^57^ ^]^ to link carbon loading of the 20 wt% MEA solution to pH. This model estimated the concentrations of all chemical species in the system as a function of pH (Figure 3b; and Section S4, Supporting Information). The chemical system had four monovalent ions: hydroxide (OH^−^) (the dominant anion in the lean solution and the anion with the highest mobility), bicarbonate (${\mathrm{HCO}}_{3}^{-}$), deprotonated carbamate (RNHCOO^−^), and protonated MEA (${\mathrm{RNH}}_{3}^{+}$). We determined that the concentration of CO_3_^2−^, the only multivalent ion, was low enough to be negligible. This simplification allowed for the application of Equation (2), which applies only to monovalent ions. We also used ^13^C nuclear magnetic resonance (NMR) spectroscopy to estimate experimentally the concentration of carbamates and carbonates as a function of carbon loading and compared these results to those from the theoretical model (Figure 3c,d; and Figure S3, Supporting Information).

The resulting concentration estimates correspond closely with the theoretical model. As ionic concentrations alone do not explain the underlying complexity of this system, we used Equation (2) to estimate the relative permselectivities of each relevant ion for each membrane (**Table** **1**; and Section S6, Supporting Information). Several properties of each ion, including factors such as size, activity, and mobility, influence the ability of that ion to diffuse across the charge‐selective membranes, resulting in varied permselectivity values.^[^ ^58^, ^59^ ^]^ For example, hydroxide has the highest ionic mobility of the ions presented, which contributes to its high permselectivity.

**Table 1:** Approximated relative permselectivity values of the relevant ions. The permselectivities of hydroxide and potassium (not shown) were set to 1.00, and all other permselectivites were calculated in reference to these values. Permselectivity values should range from 0 to 1. (Uncertainty propagated from the single membrane measurements used to calculate permselectivity, mean ± SD, n = 3)

	P^AEM^	P^CEM^
RNH3+	0.02 ± 0.01	0.94 ± 0.02
RNHCOO−	0.11 ± 0.02	0.00
HCO3−	0.57 ± 0.05	0.00
OH−	1.00	0.04 ± 0.002

When we used these permselectivity values in Equation (2) with the concentrations predicted by the theoretical model, we found that the model corresponded closely to the measured potentials (Figure 3a). With this validation of the model, we developed a computer program that predicts the *V*_oc_ of the device with any given MEA concentration and pH value (Program S1, Supporting Information).

Figure 3 demonstrates that *V*_oc_ increased with carbon loading as the difference in ion concentration between the lean and the rich solution increased. These results also revealed that the potential across the AEM contributed much less to the overall potential than that across the CEM. We accredited this finding largely to the higher [OH^−^] in the lean compartment compared to the rich compartment. Since $P_{\mathrm{OH}-}^{\mathrm{AEM}}$ was the highest permselectivity value among relevant anions, the potential established by the hydroxide gradient worked against the contribution of the other ions to significantly reduce the overall potential across the AEM. At very low carbon loading, the AEM in fact contributed negatively to the total potential of the device. At a high carbon loading, $\left[H\mathrm{CO}{}_{3}^{-}\right]$ surpassed [RNHCOO^−^], and, because${\;P}_{\mathrm{HC}O_{3}-\;}^{\mathrm{AEM}}$>${\;P}_{\mathrm{RNHCOO}-}^{\mathrm{AEM}}$, the potential contributed by the AEM increased in comparison to its contribution at lower pH values.

## 4 Evolution of Device Geometry

**Figure 4:**
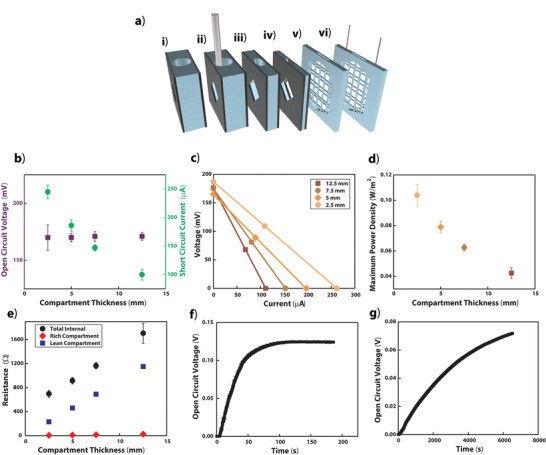
Iterations of the compartment geometry and the method of delivering CO_2_. a) The different compartment geometries used in this study: i) low‐power geometry – 10 mm thickness plus gaskets, 100 mm^2^ cross section ii) 10 mm thickness plus gaskets, 100 mm^2^ cross section with tubing for direct application of breath iii) 5 mm thickness plus gaskets, 100 mm^2^ cross section iv) 2.5 mm thickness plus gaskets, 100 mm^2^ cross section v) Increased cross‐sectional area allows for increased current using this high‐current geometry – 2.5 mm thickness with vacuum grease, 450 mm^2^ cross section vi) high‐current geometry – 2.5 mm thickness with vacuum grease, 450 mm^2^ cross section with Teflon AF 2400 tubing for the diffusion of pure CO_2_ across the tubing wall. All compartment geometries have an opening at the top for exchanging the solution and/or inserting tubing for CO_2_. b) Measured *V*_oc_ and *I*_sc_ using 20 wt% MEA and the maximally carbon loaded rich solution (pH 7.8) using varied compartment thicknesses (mean ± SD, *n* = 4 except 12.5 mm where *n* = 6). c) Representative *I*–*V* curves of cells with different total thicknesses. All lines are linear fits. d) Maximum power density as a function of total thickness calculated using *V*_oc_ and *I*_sc_ measurements in panel b). e) Total internal resistance of the device calculated using *V*_oc_ and *I*_sc_ measurements in panel b) and expected resistance of each compartment calculated using volume and conductivity measurements in Figure 2d as a function of single‐compartment thickness. Neoprene gaskets added 2.5 mm to the thickness of each compartment when used. f) Open circuit voltage over time as pure CO_2_ was added directly to rich compartments in a high‐current geometry (panel a.v.) device. g) Open circuit voltage over time as pure CO_2_ was diffused into rich compartments through the walls of Teflon AF 2400 tubing in a high‐current geometry device.

To increase the power density from the devices, we reduced the thickness of the compartments (**Figure** **4**ai,iii,iv, b–d) in order to reduce internal resistance (Figure 4e), thereby increasing the output current. We first reduced the thickness by 3D printing compartments with decreasing thicknesses: 10, 5, 2.5 mm. Then, using the thinnest compartment size, we switched from using gaskets, which contributed an additional 2.5 mm to the total thickness of each compartment, to using vacuum grease with negligible thickness to create the seal around the membrane between each compartment. Using this method, we reached a maximum power density of 0.104 ± 0.009 W m^−2^. With 0.1 mm compartments and specialized membranes, the more traditional CCRED device developed by Kim et al. reached 1 W m^−2^.^[^ ^19^ ^]^ We also created a “high‐current” geometry by increasing the cross‐sectional area of the compartment (Figure 4av; and Equation S15, Supporting Information). We noticed that osmotic pressure may cause the charge‐selective membranes to bulge, and these thin compartments with increased cross‐sectional area increased the risk of the membranes coming into direct contact, which may undermine the electric potential contributed by that compartment. To address this risk, a supportive mesh (0.5 mm diameter rods, 4 x 5.5 mm^2^ rectangular open spaces) 3D printed as part of the compartment prevented the membranes from bulging into contact. The 3D models used are accessible online (Section S11, Supporting Information).

While characterizing the device presented in this work, we often preloaded the rich solution with CO_2_ before adding it to the proper compartments. A central feature of the design presented, however, is that the device could be filled with lean solution in every compartment, and the addition of CO_2_ to every other compartment would generate useable electric power on demand. To demonstrate this concept, we examined two methods for the addition of pure CO_2_ gas directly into the rich compartments of the device: bubbling CO_2_ into the solution through an open‐ended tube (Figure 4aii) and allowing the CO_2_ to permeate through the thin walls of Teflon AF 2400 tubing (Figure 4avi). For testing these two methods, we first filled all the compartments with only lean solution, generating no initial potential. Using the high‐current geometry (Figure 4av), bubbling CO_2_ directly into the rich compartments (i.e., into every other compartment) established a *V*_oc_ of 124 mV within about 2 min (Figure 4f) from a single cell. Permeating CO_2_ across the walls of Teflon AF 2400 tubing using the same compartment geometry (Figure 4avi), however, required 2 h to reach a *V*_oc_ of ≈75 mV (Figure 4g). Although requiring CO_2_ to permeate into the solution generates a potential at a much slower rate, this method circumvents the formation of air bubbles that could contribute to internal resistance or that could become trapped within the device. This demonstration proved the viability of a RED design where an internal gas permeation process coupled with a chemical reaction establishes the ion gradient rather than the exchange of external solutions. Future designs may make it possible to increase the rate of CO_2_ addition via permeation by increasing the length of the tubing in the solution, reducing the tubing wall thickness, increasing the air pressure, or using a different specialized material.

## 5 The Power and Energy of Breath‐Established Gradients

**Figure 5:**
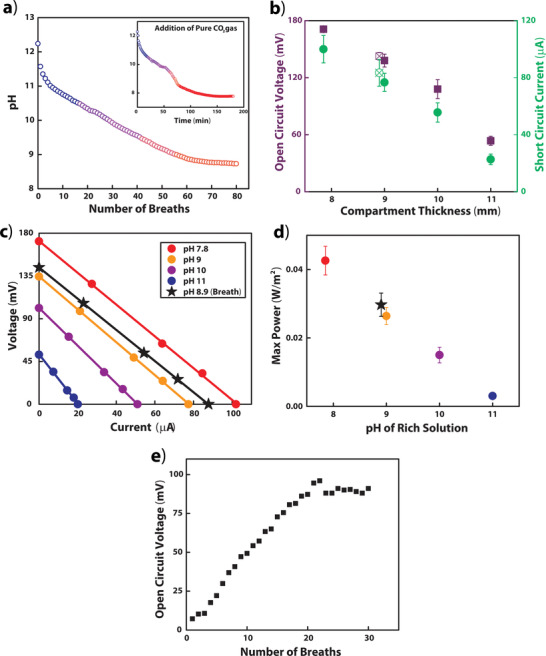
Carbon loading with breath and the resulting power output using the low‐power geometry (Figure 4ai). a) The pH change of a 10 mL, 20 wt% MEA solution with the addition of breath. Inset: The pH change over time with the slow addition of pure CO_2_ into a 20 wt% MEA solution, provided for comparison. b) Measured *V*_oc_ and *I*_sc_ using 20 wt% MEA at varied carbon loading (mean ± SD, *n* = 6 except breath where *n* = 5). The measurements of the system loaded with breath are indicated with the X on the hollow data point. c) Examples of *I*–*V* curves of device when the rich solution is loaded with breath (stars), or with pure CO_2_ to different pH values (circles). The lean solution remained at pH 12.2. All lines are linear fits. d) Maximum power density of device when loaded with breath and when loaded with pure CO_2_ to different pH values calculated using *V*_oc_ and *I*_sc_ measurements in panel b. e) Change in *V*_oc_ of one cell over time with the addition of breath directly to rich compartments in a low‐power geometry (Figure 4a ii) device.

To confirm that the CO_2_ within breath can establish the ion gradient required to generate electric power, we monitored the acidification of a 20 wt% MEA solution in response to the capture of CO_2_ from breath (**Figure** **5**a). Because the solubility of CO_2_ in a MEA solution is related to the partial pressure of CO_2_,^[^ ^60^ ^]^ the breath‐loaded rich solution only reached a lowest value of pH 8.9, ≈1 pH unit higher than the pH that could be achieved using pure CO_2_ gas. When used in the rich compartment of the device, this solution generated an *V*_oc_ of 146 mV, matching the value achieved in Figure 3a for a rich solution loaded to pH 9 using pure CO_2_ gas. We created *I*–*V* curves and calculated the maximum power density for solutions loaded with pure CO_2_ to different extents and for the solution loaded by breath (Figure 5b–d). As expected, both the current and voltage of the device improved as the carbon load increased. Again, the breath‐loaded solution with a pH of 8.9 aligned closely with the *I*–*V* curve of the pH 9 solution loaded with pure CO_2_. This agreement shows that loading the solution using breath, despite its impurities, had no detrimental impact on the power output of this device. We also did not detect any unexpected chemical species within a breath‐loaded solution using ^13^C NMR spectroscopy (Figure S3, Supporting Information).

Using the method of bubbling breath into the compartments (Figure 4aii), we demonstrated that breath added directly to the device could generate electrical potential in situ. Initially, we filled every compartment with lean solution, producing no initial potential. We supplied breath to the high ionic strength compartments to generate an ion gradient within the device and measured the potential after each breath (Figure 5e).

To demonstrate that the approach presented here could scale up to power electronic devices, we stacked cells with compartments of the high‐current geometry (Figure 4av) in series to power a LED. Fifteen cells in series using a rich solution loaded with pure CO_2_ (pH 7.8) generated an *V*_oc_ of 2.5 V and a maximum power density of 0.04 W m^−2^ cell^−1^, and successfully powered a LED. We accredited the lower maximum power density in comparison to previous measurements (Figure 4d) to the challenges of ensuring consistent conditions when using many cells (e.g., preventing bubbles that contribute to internal resistance). Using a rich solution loaded by breath (pH 8.9), 27 cells of this geometry produced an *V*_oc_ of 3.0 V and a maximum power density of 0.03 W m^−2^ cell^−1^, also successfully powering a LED (Figure 1d).

Using the concentrations approximated using NMR spectroscopy, we found that the theoretical energy density due to the change in Gibbs free energy from mixing equal quantities of lean solution and rich solution loaded with breath (pH 8.9) is ≈1.94 kJ L^−1^ mixed solution. When using a rich solution loaded with pure CO_2_ gas (pH 7.8), the theoretical energy density is ≈2.45 kJ L^−1^ mixed solution (Equation S16, Supporting Information). This corresponds to ≈53 kJ kg^−1^ CO_2_.

We measured that a single unit of the device presented in this work provided at least 0.12 J from 0.25 mL of rich solution in each of the two rich compartments (0.5 mL total) and 0.25 mL of lean solution in the lean compartment (Equation S17, Supporting Information). The theoretical change in Gibbs free energy that results from the mixing of these solutions was 1.4 J, indicating an energy efficiency of ≈8.4% for the device presented here. For reference, in previous CCRED work, Kim et al. cite an energy efficiency of 1.59%.^[^ ^19^ ^]^ For the well‐established use of saltwater and freshwater for RED, energy efficiencies commonly range from 20% to 40%.^[^ ^24^, ^26^, ^27^, ^61^, ^62^ ^]^ The greatest energetic losses (low energy conversion efficiency) came from imperfections in the selectivity of ion exchange membranes (e.g., co‐ion transport, water transport and associated osmotic pressure, and electro‐osmosis)^[^ ^63^ ^]^ and Ohmic losses (i.e., the friction losses of ion transport).^[^ ^61^, ^62^, ^63^ ^]^ In our case, the pH gradient across the membranes caused hydroxide ions to diffuse in the opposite direction with respect to the other anions, further reducing  the energy conversion efficiency of the device. We conducted an experiment in which we monitored the temperature of the lean solution of the device (20 wt% MEA, 12.5 mm thick compartments, rich solution pH 7.9) in the short circuit condition. The temperature of the solution remained at ≈24 °C for the course of the hour‐long trial. Furthermore, the membranes used in this work were designed for small ions (e.g., potassium, sodium, chloride) rather than the large, organic ions, such as carbamate, present in this system. Fumasep specifies that the CEM has a specific area resistance of 2.5–5.0 Ω cm^2^ for Na^+^, and the AEM has a specific area resistance of 5.0–9.0 Ω cm^2^ for Cl^−^ and 10–20 Ω cm^2^ for SO_4_^2−^. In contrast, we estimated that a pair of ion exchange membranes (CEM + AEM) in the presented device contributes ≈250–400 Ω cm^2^, likely because of the large, organic ions in the system and the viscosity. The use of specialized membranes and/or alternative carbon capture solvents would likely improve both the power density and energy efficiency of future iterations of this and other CCRED devices.

We recognize that the prototype presented here is only a first step toward a practical portable power source. For example, in this work, we achieved an *V*_oc_ of ≈90 mV in a single RED cell with 20 breaths, but with higher carbon capture efficiency, perhaps by using better gas distribution methods, the amount of CO_2_ in just a few breaths would likely suffice to reach the same result. Furthermore, future iterations toward a wearable power source should use a more biologically compatible carbon capture solvent. We use MEA in this work because it represents a benchmark for carbon capture solvents; however, it is also quite corrosive. Additionally, further reductions in the size of the device will not only increase the maximum power density, as explored in the previous section, but also make this technology more portable.

Finally, although this work provided a strategy for the generation of ion gradients from metabolic CO_2_, the full discharge‐recharge cycle observed in the electrocytes of electric eels has yet to be demonstrated. Applying heat to the rich solution, however, could regenerate the lean MEA solution by running the carbon capture reaction in reverse.^[^ ^64^ ^]^ To create a complete cycle using the system demonstrated in this work, the addition of CO_2_ to the device could generate the ion gradient for harvesting electric power, the potential would dissipate when applied across a load, and then heat could remove the CO_2_ to bring the device back to its original state (Figure S5, Supporting Information). Temperatures in the range of 90–110 °C, however, would be necessary to restore the MEA solution.^[^ ^64^ ^]^ We did not design the device presented in this work with such heat cycling in mind, but future work in this field could aim to complete the discharge–recharge cycle.

## 6 Conclusion

In this work, we used a metabolic waste product, CO_2_ in exhaled human breath, to generate electric power. With the design presented here, we achieved a maximum power density of 0.030 ± 0.003 W m^−2^ cell^−1^ using CO_2_ captured from breath and 0.104 ± 0.009 W m^−2^ cell^−1^ from pure CO_2_ gas. Additionally, we demonstrated that this technique could scale up to power small electronic devices, such as a LED. An optimum concentration of MEA leads to maximizing power output because the power benefit from increased charge carriers at increasing MEA concentrations is counteracted by a concomitant increase in viscosity. Of the MEA concentrations tested, we found the optimum concentration to be 20 wt% MEA. Increasing the carbon loading and reducing the compartment thickness also increased the device's power density, as supported by theory. Of the three anions that contributed significantly to the potential, we found that hydroxide had the greatest permselectivity, and that its ion gradient in the opposite direction of the other anions, in fact, subtracted from the overall potential. Additionally, the higher permeselectivity of the AEM to bicarbonate compared to carbamate indicated that bicarbonate contributed more effectively to the electric potential than carbamate.

The ability to generate the ion gradient in situ, within the RED device, using a chemical reaction rather than with a flow of solutions distinguishes this design from most previous RED devices. The use of a single uniform initial solution contributes to the ease of use and robustness of this device as well as its ability to be stored in a thermodynamically stable state, allowing future iterations of this or related devices to possibly achieve a long shelf life (Figure S8, Supporting Information). While we utilized CO_2_ in this work, other ion‐generating mechanisms could be employed to develop similar power sources. As any human user of electronic devices would continuously supply CO_2_ in breath with no special effort, the approach to power generation introduced here, with further improvements, may lend itself towards the development of noninvasively powered portable or wearable technology.

## 7 Experimental Section

### Materials

Ethanolamine, ≥ 99% (MEA), potassium hexacyanoferrate (II) trihydrate, potassium hexacyanoferrate (III), and potassium chloride were purchased from Sigma‐Aldrich (Merck‐KGaA). Fumasep FKB‐PK‐130 (cation exchange) and FAB‐PK‐130 (anion exchange) membranes were purchased from Fumatech BWT GmbH, Germany. Fumatech specifies these membranes are 100–130 µm thick, have a selectivity of 94–99%, and are stable within the pH 1–14 range. Membranes and neoprene gaskets were cut into the desired shapes using a Cricut Maker cutting machine (Cricut Inc.) The acrylic used to make the box that holds the RED components was cut using a Speedy 300 laser cutter (Trotec). The RED compartments and the screw clamp were 3D printed with a Form 2 3D printer (FormLabs) using clear resin. SGL Carbon GmbH (Germany) provided sigracell graphite battery felt, GFD, with 4.6 mm thickness. Plat‐inum wire (0.3 mm diameter) was purchased from Alfa Aesar. Water was purified to 18.2 MΩ cm with a PURELAB Flex II purifier (ELGA LabWater, Veolia).

### Device Assembly

The 3 x 3 cm^2^ compartments of varying thickness with a square 1 x 1 cm^2^ hole (Figure 4.ai,iii,iv.) were 3D printed. A high‐current geometry compartment was also 3D printed, 3.3 x 3.3 cm^2^ with 2.5 mm thickness, with a hole with a 4.5 cm^2^ cross‐sectional area and a 5.5 x 4 mm^2^ 3D printed mesh (Figure 4av). A conical opening at the top of each compartment allowed access for pipetting to add or remove solutions after assembly (Figure 4a). The box that held the compartments in series (Figure 1c) was laser cut from acrylic, and isopropanol was used to melt and weld the acrylic pieces together. A 3D printed screw clamp was epoxied at the end of the holding box to apply pressure to the stack of compartments. Membranes, AEM or CEM, with a gasket on each side separated compartments. When using the compartments with high‐current geometry (Figure 4av), Dow Corning high vacuum grease (purchased from Sigma‐Aldrich) was used instead of gaskets. Electrode assemblies were used as the first and last compartment on either end of the stack of 3D printed compartments. After the components were placed in order (Figure 1c), the screw clamp was tightened by hand. Leaks were tested for using DI water before adding MEA solutions. Compartments were rinsed twice with DI water when solutions were changed.

### Electrode Assembly

The 3 x 3 x 1.25 cm^3^ electrode compartment with a diamond shaped 1 x 1 x 1 cm^3^ cavity (Figure 1c) was 3D printed. A 3.3 x 3.3 x 7 mm^3^ electrode compartment with a cavity with a 4.5 cm^2^ cross‐sectional area to use with the high‐current geometry compartments was also printed. A conical opening on the top of each electrode compartment allowed access for adding or removing solutions by pipette as well as access for the Pt wire that was attached to the graphite felt in order to collect the current (Figure 1c). A 1 cm^2^ section, or 4 cm^2^ in the case of the high‐current geometry, of graphite felt was inserted into the electrode compartment before the assembly of the device. A CEM was used to separate the electrode compartment from the next compartment. After assembly with the other compartments, the 0.3 mm diameter platinum wires were embedded, which served as current collectors for each electrode, into the dense and thick graphite felt from the top of each compartment and held it in place with a clamp. The compartments were then filled with the electrode solution. This solution contained 0.05 m potassium hexacyanoferrate (II), 0.05 m potassium hexacyanoferrate (III), and 0.5 m potassium chloride. The redox reaction of a hexacyanoferrate solution with graphite was chosen to convert the ionic power to electric power based on previous assessment of electrode assemblies for RED done by Veerman et al.^[^ ^25^ ^]^ The electrode potential of HCF ([Fe(CN)6]^4−^/[Fe(CN)6]^3−^) is E^0^ = 0.356 V versus standard hydrogen electrode. However, because opposite reactions are occurring at the two electrodes, the Nernst potential of reduction on the cathode is counterbalanced by oxidization on the anode, resulting in a net zero contribution to the overall measured potential.^[^ ^25^ ^]^ The CEMs prevented hexacyannoferrate ions from diffusing from the electrode compartments into other compartments. The electrode solution for each new test condition was exchanged to eliminate potential offset potentials.

### 3D Printing

The 3D models were created using Microsoft 3D Builder. Components were printed using a Formlabs Form 3 stereolithography 3D printer and the Formlabs clear resin. The models of the 3D components are available online at: https://github.com/alessandroianiro/GLAM.

### Carbon Loading MEA Using Pure CO_2_

Pure CO_2_ was regulated from a pressurized tank to a pressure of 1 bar. Unless stated otherwise, CO_2_ gas was dispersed into the MEA solutions using a gas dispersion tube with a porous fritted glass tip, 4–8 µm porosity, produced by Ace Glass, Inc. and purchased from Sigma‐Aldrich. A Supelco Rotameter with a needle valve (flow range 0–110 mL min^−1^) purchased from Sigma‐Aldrich controlled CO_2_ gas flow rate. For the experiment that analyzed different MEA concentrations, CO_2_ was added until the pH stabilized, indicating maximum carbon loading. For the experiment that explored the effect of different amounts of carbon loading, CO_2_ was added at a low flow rate until the solution reached approximately the desired pH. When CO_2_ was bubbled directly into compartments of the assembled device, Tygon formula 2375 tubing (outer diameter: 1.6 mm, inner diameter: 4.8 mm) purchased from Sigma‐Aldrich was used. When CO_2_ was allowed to permeate directly into the compartments of the assembled device, Teflon AF 2400 tubing (outer diameter: 0.010 inches, inner diameter: 0.008 inches) purchased from Biogeneral (USA) was used.

### Capturing CO_2_ from Breath

For safety reasons, breath was exhaled into tubing that led to the bottom of a 10 mL falcon vial half‐filled with 5 mL of water and sealed with the exception of another tube that lead to a beaker of 20 wt% MEA solution in a fume hood. Tygon formula 2375 tubing (outer diameter: 1.6 mm, inner diameter: 4.8 mm) was used. To ensure that the water was saturated with CO_2_ so that this safety precaution would not influence the amount of CO_2_ that entered the MEA solution, breath was first extensively exhaled into just the water. Each breath, nearing the volume of the vital lung capacity of a 1.85 m tall male of average build, lasted for ≈1 min.

As the concentration of CO_2_ in the atmosphere is much lower than that in breath (around 0.04% CO_2_ in air^[^ ^65^ ^]^ compared to 3.2–6.5% CO_2_ in breath^[^ ^47^ ^]^) and the solubility of CO_2_ in MEA solutions is related to the partial pressure of CO_2_,^[^ ^60^ ^]^ it was expected that exposure of the MEA solutions to the atmosphere during experiments had a negligible impact on the overall carbon loading.

### Measurements of pH, Conductivity, and Viscosity

pH was measured using a calibrated PH8500‐SB Portable pH Meter for strong basic solutions (purchased from Apera Instruments). Conductivity was measured using a calibrated Orion 5‐Star benchtop multiparameter meter (Thermo Scientific). Viscosity was measured using an AR‐G2 rheometer (TA Instruments Ltd.) with a 1° conical plate and a 40 mm gap distance.

### Electrical Characterization

*V*_oc_ and *I*_sc_ were recorded using a Keithley 2400 SourceMeter. The potential recorded with new membranes took time to stabilize presumably due to the exchange of counterions within the membrane with different ionic species from the solution. To account for this process in the measurements, the lean and rich compartments were emptied and refilled until a consistent *V*_oc_ was measured after multiple consecutive exchanges of the solutions (typically 3–5 rinses). In this way, the membranes were “primed” so that they were not accidentally measuring erroneously high or low values during this stabilization process, which can interfere with clear interpretation of the impact of the other variables being characterized. *I*–*V* plots were constructed by connecting resistors of known value (4.62 kOhm, 1 kOhm, and 384 Ohm) one at a time in parallel to the device while monitoring the voltage across the load (i.e., the respective resistor). This voltage divider made it possible to calculate internal resistance and maximum power density of the device presented in this work (Equations S5 and S7, Supporting Information). The specific area resistance of a pair of membranes was estimated by calculating the internal resistance of the device with one cell, and then again with two cells. The one‐cell measurement and the calculated resistance of the rich and lean compartments (Equation S15, Supporting Information) were subtracted from the measured resistance of the two‐cell system, resulting in the resistance for the 1 cm^2^ area of the pair of membranes.

The potential across single membranes were measured using Ag/AgCl electrodes in a 0.5 m KCl acrylamide hydrogel. To create a hydrogel electrode, a 1 mL pipette tip was half‐filled with a solution of 2.1 m Acrylamide, 0.5 m KCl, 65.0 × 10^−3^ m *N,N’*‐Methylenebisacrylamide, and 4.4 × 10^−3^ m APS, all purchased from Sigma‐Aldrich. A Ag wire was soaked in bleach to create the Ag/AgCl wire which was then inserted into the solution filled pipette tip. Then ≈5 *μ*L of TEMED was added to cure the hydrogel solution. Once the hydrogel was solid, the tip of the pipette was cut off to expose the hydrogel. The potential of a membrane was measured with the SourceMeter by dipping these hydrogel‐Ag/AgCl electrodes into the solutions on either side of the membrane. Then the junction potential (Equation S19 and Figure S6, Supporting Information) was mathematically removed. To ensure the potential across the membrane was fully established, the solutions were exchanged on both sides of the membrane, and measurements were repeated until a stable potential was consistently measured.

### NMR Spectroscopy

^13^C NMR spectroscopy was carried out at 297.2 K on a Bruker Avance DPX 400 spectrometer at frequencies of 100.63 MHz for ^13^C nuclei. Spectra were measured in D_2_O using sodium acetate (0.5 m) as the standard and calibrated to the carbamate peak at 164.5 ppm. Data were evaluated with the MestReNova software suite (v 11.0) and all chemical shifts *δ* are reported in parts per million (ppm). The peaks were integrated, and the 0.5 m acetate standard was used to estimate the concentrations of carbamates and carbonates. The K values presented in Section S4 of the Supporting Information were used to solve for the fraction of protonated species, resulting in the final concentration estimates.

### Estimating Relative Permselectivity

Although permselectivity values change with varied conditions, like different ionic strengths, this effect was neglected so that an approximation of the preselectivity of each ion in relation to the other ions could be made. Because these values are relative, $P_{\mathrm{OH}-}^{\mathrm{AEM}}$ and $P_{K+}^{\mathrm{CEM}}$ were assigned a value of 1.00, as these permselectivities were expected to have the highest value (of the ions considered) across the respective membrane. With these values assigned, other relative permselectivities were calculated by measuring the potential across a membrane with solutions of known concentrations and applying Equation (2). MEA solutions and the ionic concentrations estimated with NMR spectroscopy were used to estimate the permselectivity values for hard to isolate ions. For example, the MEA solution carbon loaded to pH 10 was used to estimate the permselectivity of the carbamate produced in the reaction of MEA with CO_2_. The pH of each solution was measured, and the K values in Section S4 of the Supporting Information were used to calculate the protonation state of the ions.

### Estimating Energy Efficiency

The potential of the device with a 384 Ω load using the high‐current compartment geometry (Figure 4av) with 0.25 mL of rich solution in each rich compartment (0.5 mL total) and 0.25 mL of lean solution in the lean compartment was monitored. This voltage was measured for over 10 h until the potential of the device discharged completely (Figure S7, Supporting Information). The theoretical Gibbs free energy density of mixing and the energy efficiency were calculated using Equations S16, S17, and S18, Supporting Information.

### Statistical Analysis

The solutions were refreshed until a consistent *V*_oc_ was measured after consecutive exchanges of the solution. This preparation of the membranes allowed for the counterions within new membranes to exchange with counterions in the solution. All reported data were measured after this membrane preparation. The data of major novel findings are presented as the mean ± standard deviation (SD). The sample size for any given data set ranges from *n* = 3 to *n* = 6 and is reported in the respective figure caption. Uncertainty was propagated accordingly when measured values were used in calculations.

## Conflict of Interest

The authors declare no conflict of interest.

## Author Contributions

T.J.K., A.G., and M.M. conceived the project. A.I. and M.M. supervised the work. T.J.K., A.G., D.N., A.I., and M.M. designed the experiments. A.I. created the theoretical model and the associated script. T.J.K., A.G., D.N., and A.I. contributed to data analysis. P.B.V.S. collected NMR spectroscopy data and provided the corresponding figures. T.J.K. and D.N. collected all other data and developed the device design. A.C. suggested the use of MEA and provided guidance. All authors contributed to the final manuscript.

## Supporting information

Supporting Information

## Data Availability

The data that support the findings of this study are available from the corresponding author upon reasonable request.
