# Supplementary material for: The Green Lean Amine Machine: Harvesting Electric Power While Capturing Carbon Dioxide from Breath
Source: Adv Sci (Weinh). 2021 May 28;8(15):2100995. doi: 10.1002/advs.202100995 (PMC8336495; doi:10.1002/advs.202100995)
Supplement: Supplementary file 1 — Supporting Information [file ADVS-8-2100995-s001.pdf]

WILEY-VCH

© 2021 Wiley-VCH GmbH

ADVANCED SCIENCE
Open Access

Supporting Information

for *Adv. Sci.,* DOI: 10.1002/advs.202100995

The Green Lean Amine Machine: Harvesting electric power while capturing carbon dioxide from breath

Trevor J. Kalkus, Anirvan Guha, Philip B.V. Scholten, Dmitrii Nagornii, Ali Coskun, Alessandro Ianiro, and Michael Mayer**

Supporting Information

The Green Lean Amine Machine: Harvesting electric power while capturing carbon dioxide from breath

Trevor J. Kalkus, Anirvan Guha, Philip B.V. Scholten, Dmitrii Nagornii, Ali Coskun, Alessandro Ianiro, Michael Mayer**

S1. Voltage increased linearly with cells added in series

Similar to stacking batteries in series, adding RED unit cells in series linearly increases the voltage of a RED stack. In Figure S1, we demonstrate that the power source presented in this work follows this principle. We used the low-power geometry compartments (Figure 4ai) and a rich solution at pH 9 for this demonstration.

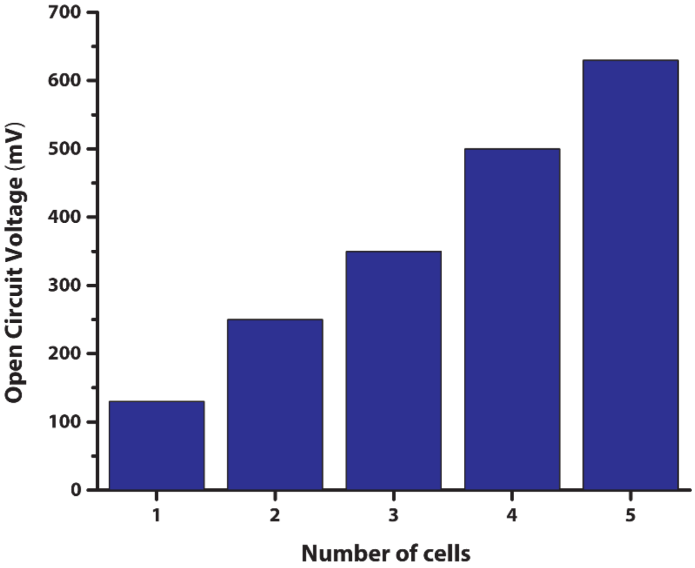

Figure S1. The open circuit voltage of the CCRED device with varied numbers of RED unit cells.

S2. Calculating internal resistance and maximum power density

The relationship between the power, voltage, current, and resistance of a system is represented by the following equations:[1]

$$P = I \times V \quad \text{(S1)}$$

$$I = \frac{V}{R} \quad \text{(S2)}$$

$$P = \frac{V^2}{R} \quad \text{(S3)}$$

where P is power (W), V is voltage (V), I is current (A), and R is resistance (Ω). The maximum power transfer theorem states that a DC power source provides maximum power when resistance of the load (R_L) is equal to the internal resistance (R_{int}) of the power source.[1,2] For a power source with a linear I-V relationship, this condition is met with the voltage across a load (V_L) equals half the V_{oc}.[1,2] To calculate R_{int}, a R_L of known value was connected to the power source in series to create a voltage divider. Using the measured V_L, the internal resistance could be calculated using the following equation:

$$V_L = V_{OC} \frac{R_L}{R_L + R_{int}} \tag{S4}$$

Alternatively, because of the linear nature of the I-V curve, we could solve for the internal resistance (R_{int}) of the device using the short circuit current (I_{sc}):

$$R_{int} = \frac{V_{oc}}{I_{sc}} \tag{S5}$$

Knowing that maximum power (P_{max}) results when $R_L = R_{int}$ and $V_L = V_{oc}/2$, we can solve for P_{max} using known values:

$$P_{max} = \frac{{V_{OC}}^2}{4R_{int}} \tag{S6}$$

We solved for maximum power density ($\rho_{P\ max}$) by dividing this result by cross-sectional area (A, m^2) and number of cells (N):

$$\rho_{P\ max} = \frac{{V_{OC}}^2}{4ANR_{int}} \tag{S7}$$

S3. Deionized water as low ionic strength solution

In the previous CCRED work, Kim *et al.* used deionized (DI) water as the low ionic strength solution.[3] The lean 20 wt.% MEA solution had a conductivity of approximately 1.1 mS cm^{-1} compared to DI water with a conductivity of 5.5×10^{-5} mS cm^{-1}. Works on RED often cite the low ionic strength compartment as the highest contributor to internal resistance.[4] Equation S7 illustrates how maximum power density increases due to decreased internal resistance. To illustrate the impact of using lean solution instead of DI water, we

performed a demonstration with water as the low ionic strength solution in the low-power compartment geometry (Figure 4ai) and the 20 wt.% MEA rich solution at pH 7.8 as the high ionic strength solution (Figure S2). Although the V_{oc} was marginally higher (DI water:200 mV, lean solution: 170mV) when using DI water rather than lean solution due to the greater difference in ionic concentrations, the maximum power density when using the lean solution was approximately six times higher than when using DI water due to a reduced internal resistance. It should be noted that the magnitude of this difference will diminish as the low ionic strength compartment is made thinner, because the compartment's contribution to the total internal resistance of the device will also be diminished. The use of lean solution as the low ionic strength solution also contributes to the elegance of this design, as discussed in the main text, and allows for completely closed system designs (Figure S5). The use of DI water requires a constant supply of DI water, which would then require special disposal protocols due to the contamination of carbon capture reagents.

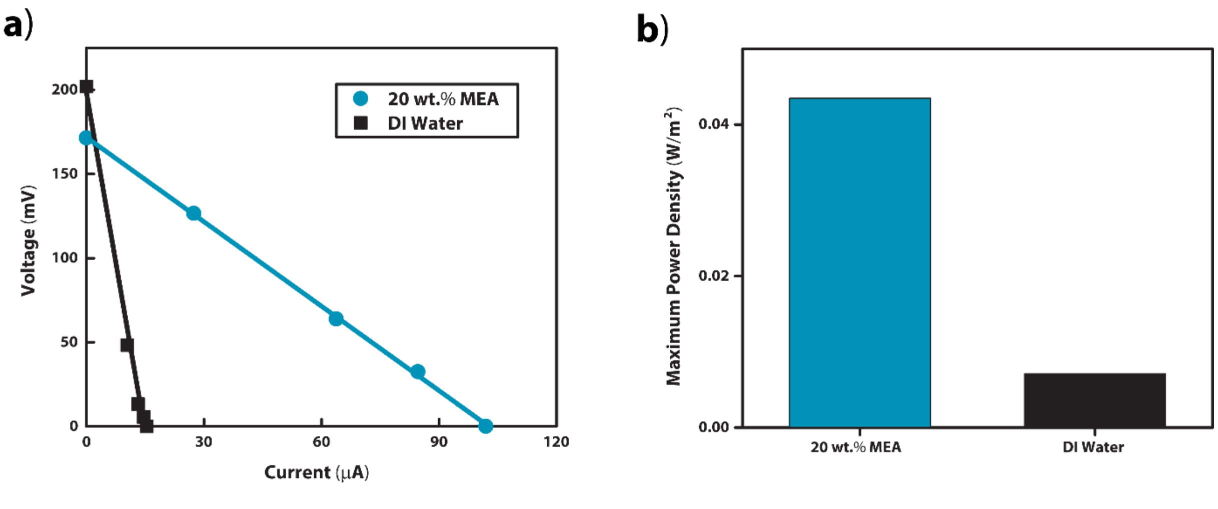

Figure S2. Analysis of power when using DI water or lean MEA solution as the low ionic strength solution when using the low-power compartment geometry. **a)** Current-voltage plot of the two different conditions. All lines are linear fits. **b)** Maximum power density of the two conditions.

S4. Modelling ionic concentrations

To estimate the concentrations of the ionic species in the system, which are necessary to predict the potential across each membrane and the total open circuit voltage using

Equation 2, we modified the theoretical model proposed by McCann et al.[5] According to this model, the equilibrium concentration of the ionic species in a MEA solution upon addition of CO_2 is described by the following set of equations:

$$K_1 = \frac{[H_2CO_3]}{\quad} \qquad K_4 = \frac{[H_2CO_3]}{\quad} \qquad K_7 = \frac{[RNHCOOH][H_2O]}{\quad}$$
$$K_2 = \frac{[HCO_3^-]}{\quad} \qquad K_5 = \frac{[RNH_3^+]}{\quad} \qquad K_8 = \frac{[RNHCOO^-][H_2O]}{\quad} \qquad K_{10} = \frac{[RNHCOOH]}{\quad} \qquad \text{(S8)}$$
$$K_3 = \frac{[HCO_3^-]}{\quad} \qquad K_6 = \frac{[H_2O]}{\quad} \qquad K_9 = \frac{[RNHCOOH]}{\quad}$$

This set of equations can be solved as a function of pH and the total initial MEA concentration ($[MEA]_0$) by imposing the constraints of nitrogen balance and charge neutrality:

$$[RNH_2] + [RNH_3^+] + [RNHCOO^-] + [RNHCOOH] = [MEA]_0 \qquad \text{(S9)}$$
$$[RNH_3^+] + [H^+] = \left[HCO_3^-\right] + [OH^-] + 2[CO_3^{2-}] + [RNHCOO^-] \qquad \text{(S10)}$$

We chose pH as a reaction coordinate because we can easily measured it experimentally. To solve this system of algebraic equations we used the ***nonlinsolve*** function of the *Sympy* module of Python, allowing us to predict the concentration of all species from pH, $[MEA]_0$ and the values of the equilibrium constants $K_1 - K_{10}$.

We evaluated the resulting expressions for $7.5 < pH < pH_{\text{lean}}$ to provide the concentration of all ionic species as a function of the carbon loading, where pH_{lean} (i.e. the pH of the MEA solution when no CO_2 is captured) is given by

$$pH_{\text{lean}} = -\log_{10}\left[\frac{2}{K_6\left(\frac{K_5}{K_6}+\sqrt{\frac{K_5}{K_6}}\sqrt{\frac{K_5}{K_6}+4[MEA]_0}\right)}\right] \qquad \text{(S11)}$$

Subsequently, the values of the equilibrium constants have been refined as follows. First, we corrected the constants for temperature because the values reported by McCann *et al.*[5] were obtained at 30°C. We assumed that the temperature dependence of the constants is given by

$$K_i = e^{\frac{\Delta G_0^j}{RT}}, \qquad \text{(S12)}$$

where ΔG_0^j is the standard Gibbs energy of the reaction j. It follows that

$$K_i(T_2) = e^{\frac{T}{T_2}\log[K_i(T)]}, \quad \text{(S13)}$$

with $T = 303.15$ K, $T_2 = 293.15$ K. We then developed a fitting algorithm to fit the theoretical concentration of carbonates ($[carbonates] = \left[H\,CO_3^-\right] + [H_2CO_3] + [CO_3^{2-}]$) and carbamates ($[carbamates] = [RNHCOO^-] + [RNHCOOH]$) to the experimental NMR data. The algorithm optimizes the value of K_1, K_2, K_7, K_8, K_9, while the other constants, describing protonation-deprotonation equilibria, are not fitted because the concentrations determined via NMR are the sum of protonated and deprotonated species.[6] The concentrations of carbonates and carbamates are fitted simultaneously by concatenating them in a one-dimensional array. A non-linear least squares fitting was performed using the Nelder Meads method implemented in the *lmfit* library of Python. The resulting optimized constants are reported below:

$K_1 = 0.0009988$
$K_2 = 60499391.63$
$K_3 = 43753705451.36$
$K_4 = 3203.61$
$K_5 = 4666049517.45$

$K_6 = 200342057809765.0$
$K_7, = 278249.57$
$K_8, = 15.38$
$K_9 = 196.47$
$K_{10}, = 55654539.92$

The refined model was used to compute the concentrations of all species in solution as a function of pH, as shown in Figure 3b. This model can be accessed using Script S1.

S5. Nuclear magnetic resonance spectroscopy

Figure 3c in the main text shows the segment of the NMR spectroscopy traces that we used to estimate carbamate and bicarbonate concentration. Figure S3 shows the complete NMR spectroscopy traces, including the NMR spectroscopy trace for the solution carbon loaded with breath. The lack of unexpected peaks supports that, despite the impurities found

in human breath, we were unable to detect the formation of alternative chemical species using ^{13}C NMR spectroscopy.

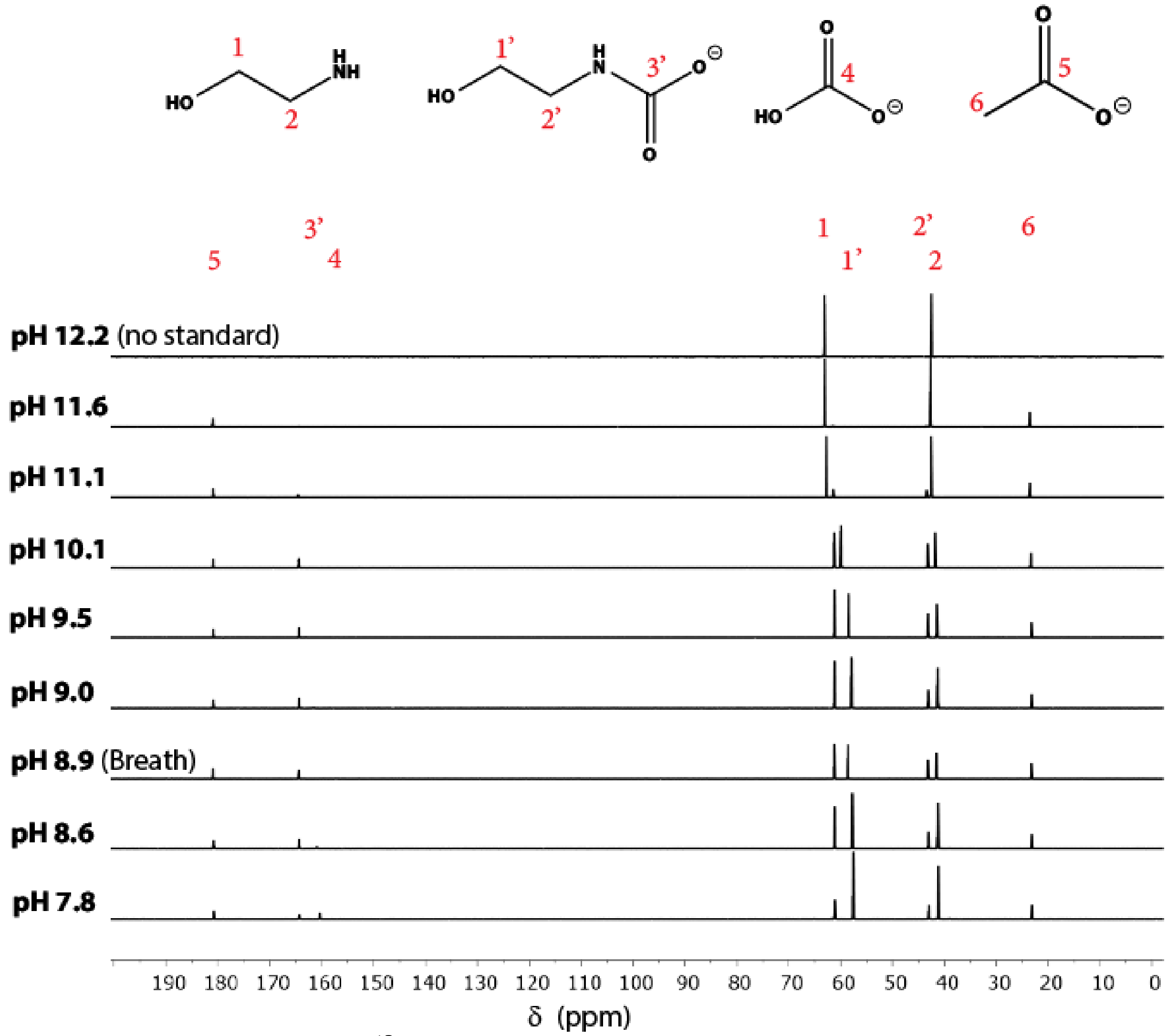

Figure S3. The complete traces of ^{13}C NMR spectroscopy measurements. We labelled the peaks in the traces with the same numbers and the carbons in the drawn structures (from left to right: MEA, protonated MEA, bicarbonate, acetate (used as a standard at 0.05 M)) to illustrate which carbons correspond to which peaks.

S6. Estimating relative ion permselectivities

By rearranging Equation 2 in the main text, the permselectivity value of an individual ion for a membrane can be solved in relation to the permselectivities of the other ions for the same membrane. Here we use the example of potassium hydroxide inside (i) and outside (o) of a membrane:

$$\frac{P_{K+}^{M}}{P_{OH-}^{M}} = \frac{e^{\frac{V_M F}{RT}}[OH^-]_o - [OH^-]_i}{e^{\frac{V_M F}{RT}}[K^+]_i - [K^+]_o} \qquad \text{(S14)}$$

We set P_{K+}^{CEM} and P_{OH-}^{AEM} to 1 and solved for P_{K+}^{AEM} and P_{OH-}^{CEM} by applying Equation S14 with the measurements of single membrane potentials produced by different concentrations of KOH. Similarly, we solved for the relative permseletivity of other ions. We measured the potential across a single membrane using solutions where only one ion's relative permselectivity value was not known. To solve for $P_{RNHCOO-}^{AEM}$, for example, we used the V_{AEM} measurement at pH 10 and the concentrations estimated by NMR spectroscopy. After solving the relative permselectivities of the each ion, we then applied Equation 2 and the other concentrations found with NMR spectroscopy to calculate the expected membrane potentials at other pH values. The purpose of this calculation was to ensure that the approximated permselectivity values remained valid in different ionic conditions (i.e., varied carbon loadings). The results of these calculations corresponded closely with the actual measurements (Figure S4).

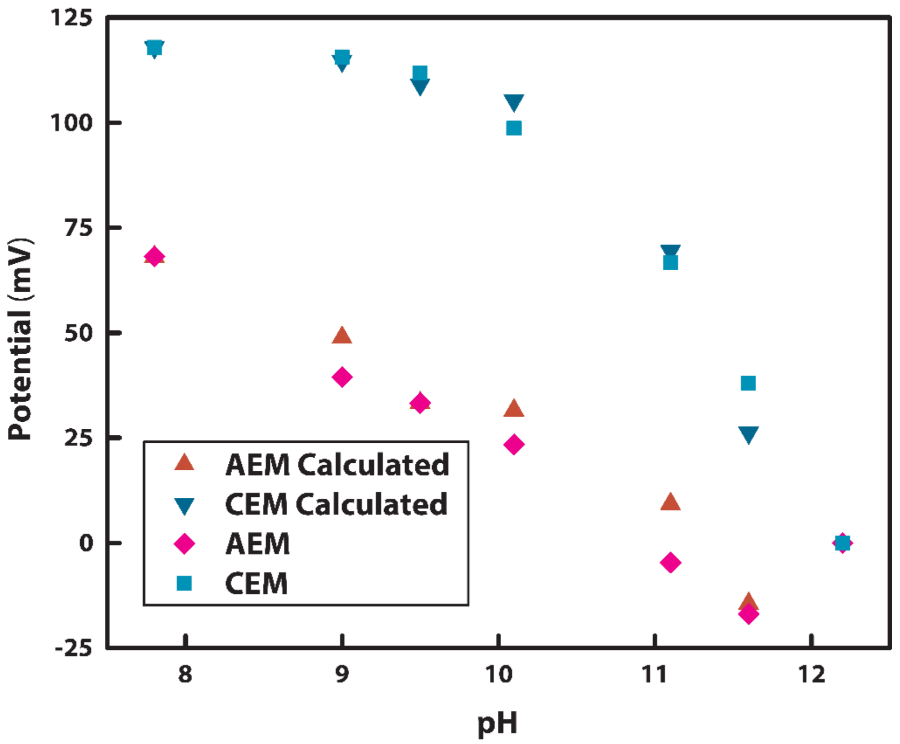

Figure S4. The measured V_{oc} of each membrane (corrected for the junction potential) compared to the expected V_{oc} of each membrane when calculated using the approximated permselectivities and concentrations estimated using NMR spectroscopy.

S7. Calculating compartment resistance

We calculated the resistance contributed by a volume of liquid by

$$R = \frac{L}{A\kappa} \tag{S15}$$

where L is the length (i.e., compartment thickness) (m), A is the cross-sectional area (m^2), and κ is the conductivity (S). Increasing the cross-sectional area and decreasing the compartment thickness results in the decreased resistance of the compartment, thereby increasing the current and the power output of the device (Equation S2 and S3).

S8. Estimating energy density

Equation S16 describes the Gibbs free energy density that results from creating a mixture (mix) from a low salt solution (LSS) and a high salt solution (HSS):[7]

$$\frac{\Delta G_{\text{mix}}}{v_{\text{mix}}} \approx RT\{f\,[\sum_i c_i \ln(\gamma_i \bar{c}_i)]_{\text{LSS}} + (1-f)[\sum_i c_i \ln(\gamma_i \bar{c}_i)]_{\text{HSS}} - [\sum_i c_i \ln(\gamma_i \bar{c}_i)]_{\text{mix}}\} \tag{S16}$$

where γ_i is the activity coefficient of ion i, c_i is the concentration (M) of ion i, f is the volume fraction of the LSS to the total mixed solution (i.e., $f = v_{\text{LSS}}/(v_{\text{LSS}} + v_{\text{HSS}}) = v_{\text{LSS}}/v_{\text{mix}}$) and $\bar{c}_i = c_i/(1 \cdot \text{mol} \cdot \text{L}^{-1})$.

We solved for the measured energy produced by the device using:

$$E = \int_0^t P\, dt \tag{S17}$$

where E is energy (J) and t is time (s). We measured the voltage across a resistor and solved for P using Equation S3. We then calculated energy efficiency using:

$$e_E = \frac{E}{\Delta G_{mix}} \tag{S18}$$

S9. A possible discharge-recharge cycle for the CCRED device presented

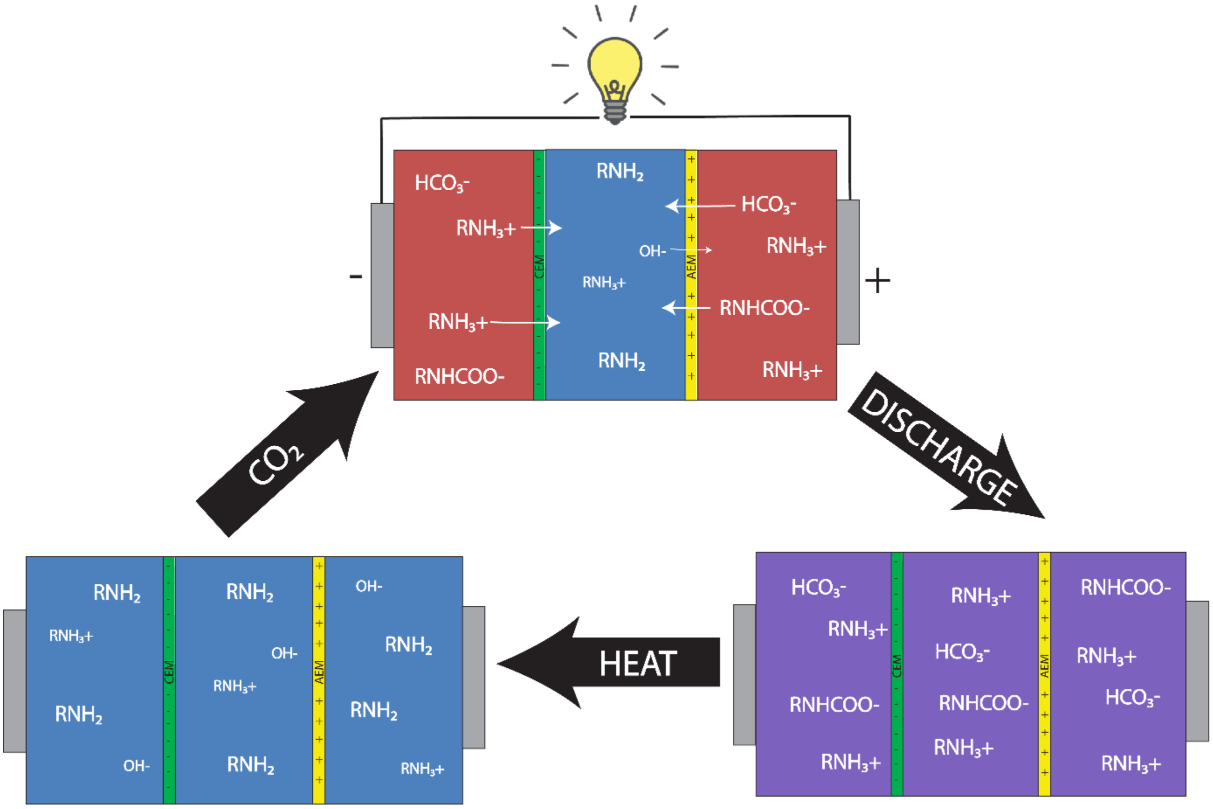

Figure S5. A diagram demonstrating the possibility of cycling the CCRED device presented through discharge-recharge cycles by using heat to return a discharged device to the initial state.

S10. Calculating and removing junction potential

Junction potentials arise due to differences in ion mobility and ionic selectivity of a separator (e.g. the edge of a hydrogel).[8] When we used hydrogel electrodes to measure the potential of a single ion exchange membrane, the solutions on either side of the membrane consisted of different ions and/or different concentrations of the same ions. The way these ions diffused in or out of the hydrogel electrodes resulted in different junction potentials on each electrode. To remove this artifact, we calculated the junction potentials using

$$V_j = \frac{\sum_i \frac{|z_i|u_i}{z_i}[a_i(2) - a_i(1)]}{\sum_i |z_i|u_i[a_i(2) - a_i(1)]} \frac{RT}{F} \ln \frac{\sum_i |z_i|u_i a_i(1)}{\sum_i |z_i|u_i a_i(2)} \quad \text{(S19)}$$

where z_i is the valence charge of ion i, a_i is the activity of ion i for solution 1 and solution 2, and u_i is the electrophoretic mobility of ion i.[8] We approximated the activity coefficient using the Debye-Huckel equation to estimate the activity of each ion:[9]

$$\gamma_i(x) = \exp\left[\frac{0.51 z_i^2 \sqrt{I(x)}}{1+(\frac{A_i}{305})\sqrt{I(x)}}\right] \quad \text{(S20)}$$

$$a_i = \gamma_i \, c_i \quad \text{(S21)}$$

where A_i is the effective hydrated ion radius of ion i, I is the ionic strength of solution x, and c is the concentration of ion i. We illustrate the difference between initial measurements and the data after corrected for junction potential in Figure S6.

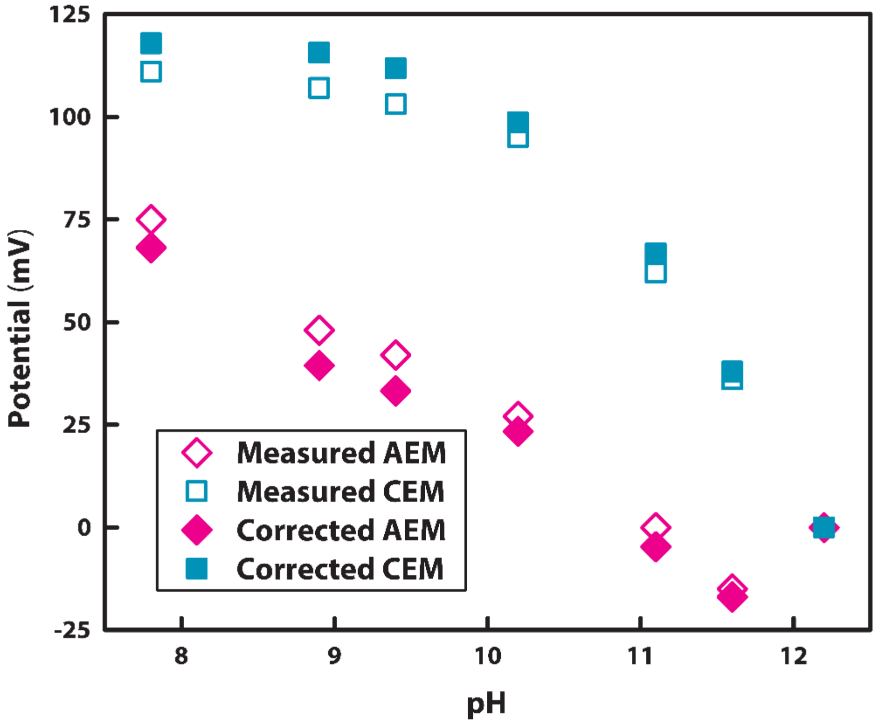

Figure S6. The hollow data points show the raw measurements of V_{oc} across a CEM and an AEM. The solid data points show the data corrected for junction potential.

S11. Instructions to run supplementary computer program and download 3D models

We created a computer program, Program S1, for predicting the V_{oc} of the device and of each membrane for a range of pH values of the rich solution when provided the MEA concentration, temperature, and number of cells in series. The computer program can be downloaded at the following address: https://github.com/alessandroianiro/GLAM. To run the program, first install the latest Anaconda distribution (https://www.anaconda.com/products/individual).

The 3D models of the compartments that were 3D printed and used in this work can also be downloaded at the same address: https://github.com/alessandroianiro/GLAM. These models can be viewed using any 3D rendering software. We designed these models in Microsoft 3D Builder.

S12. Depletion of the power of the device

The voltage provided by the device across a 384 Ω load was monitored until the potential was almost completely depleted (Figure S7). We used the high-current compartment geometry (Figure 4av) with 0.25 mL of rich solution in each rich compartment (0.5 mL total) and 0.25 mL of lean solution in the lean compartment. The combination of the high-current geometry, the low resistive load, and the small amount of solution aided in rapid depletion.

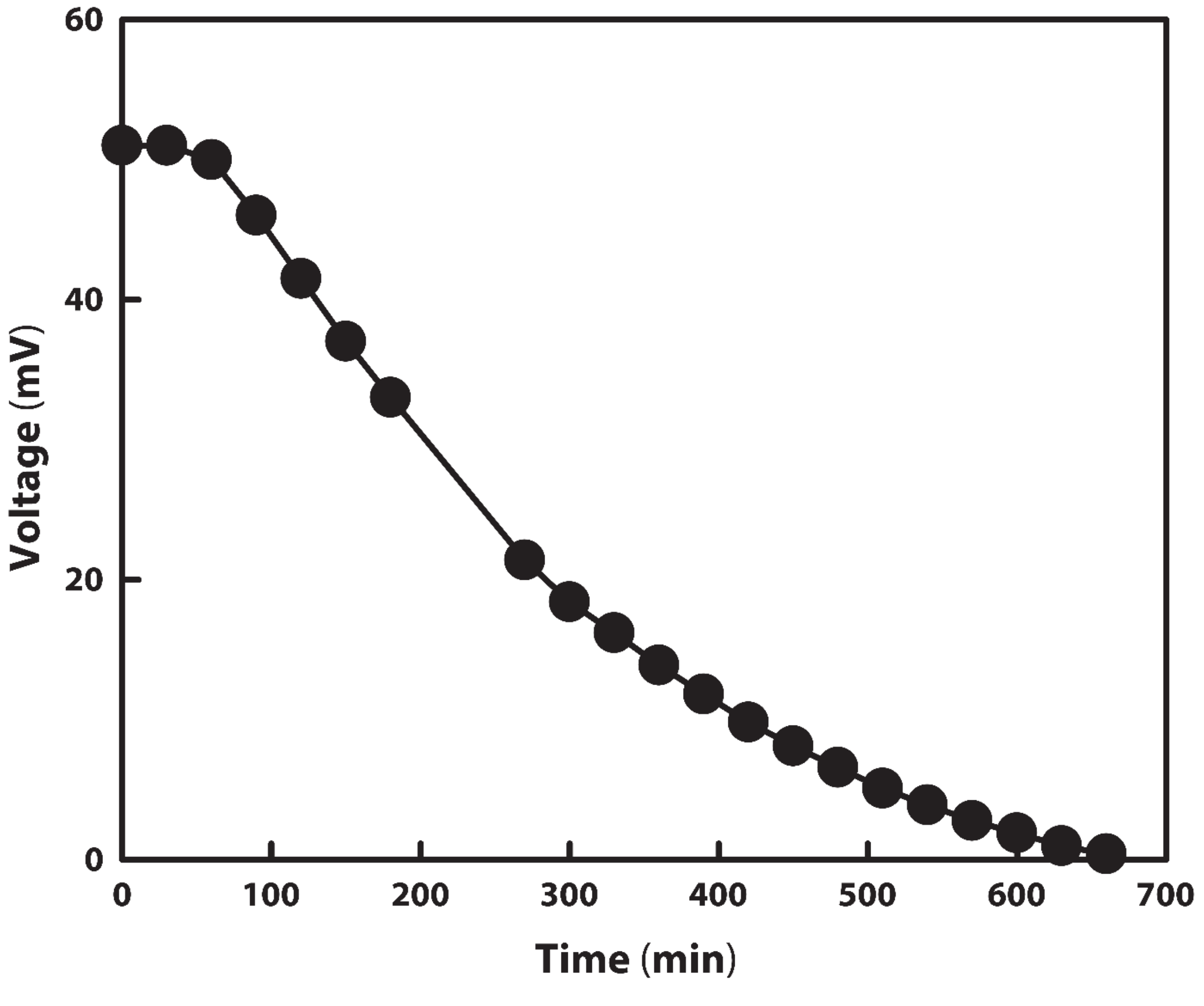

Figure S7. The depletion of the power of the device over time (high-current geometry, 20 wt.% MEA, rich solution pH 7.8, 0.25 mL of solution in each MEA compartment)

S13. Stability of device performance after storage time in lean MEA

The device design presented in this work requires only one initial MEA solution, providing simplicity for the user. Because of this design, the device could be stored with just the lean solution filling all the compartments, ready to be activated by the breath of the user. Unlike many power sources where the potential is already stored in the device, there is no concern for the dissipation of the power of the device during storage because the user provides the necessary ion gradients at the time of use. We wanted to characterize, however, the impact of storage with lean MEA solution on the performance of the device (Figure S8). Over the course of five days, the device was stored with the electrode solution in the electrode compartments and the lean 20 wt.% MEA in all other compartments. Every 24 hours, we removed the lean solution used for storage and filled the lean and rich compartments with lean and rich solution respectively. Then we measured the V_{oc} and I_{sc} before emptying the compartments and filling them all with lean solution again to be stored for another day. This allowed us to characterize the same setup after each day of storage. A small daily decrease of V_{oc} and I_{sc} was observed. It is possible that increased swelling or slow degradation of the membranes due to the long exposure to the solution diminished the membranes' charge selectivity. For the future development of this or related devices, further investigation into the cause of the decreased performance after storage could help inform and improve the design to be more stable over time.

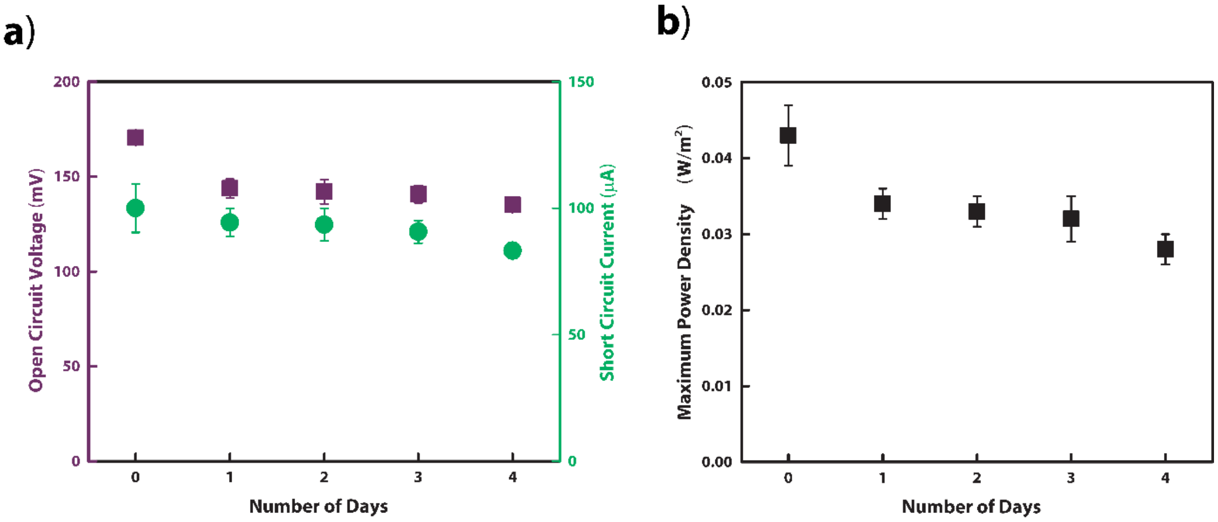

Figure S8. The performance of the device (20 wt.% MEA, 12.5 mm compartment size, rich solution pH 7.8) every 24 hours after storage with lean MEA solution. **a)** The V_{oc} and I_{sc} of the device measured each day (mean ± SD, n = 3 except Day 0 where n = 6). **b)** Maximum power density of the device each day calculated using V_{oc} and I_{sc} measurements in panel a.

References:

[1] S. N. Makarov, R. Ludwig, S. J. Bitar, *Practical Electrical Engineering*, Springer, **2016**.

[2] T. B. H. Schroeder, A. Guha, A. Lamoureux, G. VanRenterghem, D. Sept, M. Shtein, J. Yang, M. Mayer, *Nature* **2017**, *552*, 214.

[3] H. Kim, Y.-E. Kim, N.-J. Jeong, K.-S. Hwang, J.-H. Han, J.-Y. Nam, E. Jwa, S.-C. Nam, S.-Y. Park, Y.-I. Yoon, C.-S. Kim, *J. CO2 Util.* **2017**, *20*, 312.

[4] J. Veerman, J. W. Post, M. Saakes, S. J. Metz, G. J. Harmsen, *J. Membr. Sci.* **2008**, *310*, 418.

[5] N. McCann, D. Phan, X. Wang, W. Conway, R. Burns, M. Attalla, G. Puxty, M. Maeder, *J. Phys. Chem. A* **2009**, *113*, 5022.

[6] A. D. Wilson, F. F. Stewart, *RSC Adv.* **2014**, *4*, 11039.

[7] N. Y. Yip, M. Elimelech, *Environ. Sci. Technol.* **2012**, *46*, 5230.

[8] R. S. Kingsbury, S. Flotron, S. Zhu, D. F. Call, O. Coronell, *Environ. Sci. Technol.* **2018**, *52*, 4929.

[9] R. Long, B. Li, Z. Liu, W. Liu, *Energy* **2018**, *151*, 1.
